# Prediction of anticancer molecules using hybrid model developed on molecules screened against NCI-60 cancer cell lines

**DOI:** 10.1186/s12885-016-2082-y

**Published:** 2016-02-09

**Authors:** Harinder Singh, Rahul Kumar, Sandeep Singh, Kumardeep Chaudhary, Ankur Gautam, Gajendra P. S. Raghava

**Affiliations:** Bioinformatics Centre, Institute of Microbial Technology, Sector 39-A, Chandigarh, India

**Keywords:** Cancer inhibitors, Classification of cancer inhibitors and non-inhibitors, Active substructure, Active functional groups, Fingerprints, QSAR, Potency score, SVM light

## Abstract

**Background:**

In past, numerous quantitative structure-activity relationship (QSAR) based models have been developed for predicting anticancer activity for a specific class of molecules against different cancer drug targets. In contrast, limited attempt have been made to predict the anticancer activity of a diverse class of chemicals against a wide variety of cancer cell lines. In this study, we described a hybrid method developed on thousands of anticancer and non-anticancer molecules tested against National Cancer Institute (NCI) 60 cancer cell lines.

**Results:**

Our analysis of anticancer molecules revealed that majority of anticancer molecules contains 18–24 carbon atoms and are dominated by functional groups like R_2_NH, R_3_N, ROH, RCOR, and ROR. It was also observed that certain substructures (e.g., 1-methoxy-4-methylbenzene, 1-methoxy benzene, Nitrobenzene, Indole, Propenyl benzene) are more abundant in anticancer molecules. Next, we developed anticancer molecule prediction models using various machine-learning techniques and achieved maximum matthews correlation coefficient (MCC) of 0.81 with 90.40 % accuracy using support vector machine (SVM) based models. In another approach, a novel similarity or potency score based method has been developed using selected fragments/fingerprints and achieved maximum MCC of 0.82 with 90.65 % accuracy. Finally, we combined the strength of above methods and developed a hybrid method with maximum MCC of 0.85 with 92.47 % accuracy.

**Conclusions:**

We developed a hybrid method utilizing the best of machine learning and potency score based method. The highly accurate hybrid method can be used for classification of anticancer and non-anticancer molecules. In order to facilitate scientific community working in the field of anticancer drug discovery, we integrate hybrid and potency method in a web server CancerIN. This server provides various facilities that includes; virtual screening of anticancer molecules, analog based drug design, and similarity with known anticancer molecules (http://crdd.osdd.net/oscadd/cancerin).

**Electronic supplementary material:**

The online version of this article (doi:10.1186/s12885-016-2082-y) contains supplementary material, which is available to authorized users.

## Background

One of the major challenges in the field of drug discovery is to design effective drugs against cancer. Existing drugs have their limitations that includes, side effects of drugs, high toxicity, drug resistance towards current anticancer drugs [1]. There is a pressing need to improve the drug arsenal to fight against this deadly disease. Experimental techniques used for drug discovery are costly and time-consuming. Thus, there is a need to develop in silico techniques for designing anticancer drugs.

In the past, attempts have been made to develop computational methods to design/predict anticancer molecules. Recently, various studies modelled the drug behaviour against multiple cancer cell lines using different genomics features. Based on the genomic data i.e., DNA copy number, gene expression, mutations and methylation the drug sensitivity is predicted. Either single gene features predict the drug sensitivity or multigene features [2–9]. In spite of advances in genomics, modelling the behaviour of thousands of drug is still a challenging task. The other approach is quantitative structure-activity relationship (QSAR) based models, where chemical features are used to predict inhibitors against specific cancer drug targets [10–18]. Most of the QSAR-based models have been developed for predicting inhibition activity of a specific class of molecules against a given drug target [19–23]. Recently, QSAR-based models have been developed for inhibition activity prediction of any class of molecule (irrespective of molecules class) against cancer drug target EGFR [24]. In contrast, limited attempt have been made to develop methods for predicting the anticancer activity of molecules against cancer cell lines. Kumar et al. developed one such method against 16 pancreatic cancer cell lines, which consider cancer cell as a whole for the anticancer activity irrespective of drug targets [25].

Development Therapeutics Program (DTP) stores thousands of molecules tested against NCI-60 human cancer cell lines [26]. Researchers have exploited this massive dataset for various studies like a prediction of anticancer molecules. Josefin and coworkers showed that molecules with similar activity profiles or structure often show similar mode of action (MOA) [27]. Recently, Li et. al. have developed a method called CDRUG [28], for predicting the potential anticancer molecules using the NCI-60 data. They developed similarity-based approach using relative frequency-weighted fingerprints, Tanimoto coefficient, and MinMax Kernel and achieved area under the curve (AUC) value of 0.88. CDRUG is based upon thousands of fingerprints generated using jCompoundMapper [29] and offers little understanding of the algorithm. Further, JCompoundMapper package generates only chemical graph fingerprints with no substructure-based fingerprint. In this study, a systematic attempt has been made to develop a method for predicting anticancer molecules. Here, we have used a large dataset containing 8565 anticancer and 9804 non-anticancer molecules obtained from NCI-60 [28]. Using this large dataset, we identify important fingerprints/substructures that play a significant role in the classification of anticancer and non-anticancer molecules. We developed a hybrid method by combining the machine learning and similarity-based method developed on the above dataset for classification of anticancer and non-anticancer molecules.

## Methods

### Dataset

Dataset used in this study was taken from Li and Huang study [28], which consists of 8565 anticancer and 9804 non-anticancer molecules. This dataset is compiled from the NCI-60 DTP project, and it is available at http://bsb.kiz.ac.cn/site_media/download/CDRUG/Benchmark.rar. In NCI-60 DTP project, two-stage screening of molecules was carried out. In the first stage, all the molecules were screened on 60 cell lines at 10^−5^ molar (15 μg/ml). Molecules showing significant growth inhibition were further tested on NCI-60 at five different concentrations. The results of screening were analyzed by NCI COMPARE algorithm [30].

### Fingerprint calculation

PaDEL software [31] was used to calculate fingerprints, which calculates ten types of fingerprints viz. CDK, Estate fingerprints, MACCS fingerprints, PubChem fingerprints, substructure fingerprint and Klekota-Foth fingerprints and their respective counts. The details about PaDEL package and different fingerprints are available at PaDEL website.

### Fingerprint or feature selection

In this study, we used an MCC-based approach for feature selection, where mean of each fingerprint in active and inactive dataset was calculated using the eqs. 1 and 2 [32].

1 $$ {\mathrm{F}}_{\mathrm{i}}^{\mathrm{A}}=\frac{{\displaystyle {\sum}_{\mathrm{j}=1}^{\mathrm{NA}}}{\mathrm{D}}_{\mathrm{i}}^{\mathrm{j}}}{\mathrm{NA}}\cdot $$

2 $$ {\mathrm{F}}_{\mathrm{i}}^{\mathrm{I}}=\frac{{\displaystyle {\sum}_{\mathrm{j}=1}^{\mathrm{NI}}}{\mathrm{D}}_{\mathrm{i}}^{\mathrm{j}}}{\mathrm{NI}} \cdot $$

Where F_i_^A^ and F_i_^I^ represent mean of i^th^ fingerprint in active (A) and inactive (I) molecules respectively. NA and NI is the number of molecules in active and inactive datasets respectively. D_i_^j^ is the value of i^th^ fingerprint for the j^th^ molecule (value is either 0 or 1). For active molecules, j varies from 1 to NA and for inactive molecules j varies from 1 to NI. Next, we classify the anticancer and non-anticancer molecules based on the compound score (C_score_) of a single fingerprint. If the value of fingerprint is 1, C_score_ is the difference between F_i_^A^ and F_i_^I^, else the C_score_ is the difference between F_i_^I^ and F_i_^A^. Following equation was used to calculate C_score_

3 $$ {\mathrm{C}}_{\mathrm{score}}^{\mathrm{j}}=\left\{\begin{array}{c}\hfill {\mathrm{F}}_{\mathrm{i}}^{\mathrm{A}}-{\mathrm{F}}_{\mathrm{i}}^{\mathrm{I}},\ \mathrm{if}\ {\mathrm{D}}_{\mathrm{i}}=1\hfill \\ {}\hfill {\mathrm{F}}_{\mathrm{i}}^{\mathrm{I}}-{\mathrm{F}}_{\mathrm{i}}^{\mathrm{A}},\ \mathrm{if}\ {\mathrm{D}}_{\mathrm{i}}=0\hfill \end{array}\right. $$

Where C^j^_score_ is a compound score of the j^th^ molecule for i^th^ fingerprint. Each molecule is having, C_score_ more than threshold was classified as active, otherwise classified as inactive molecule. This technique was repeated for each fingerprint at the different threshold. Finally, the performance of each fingerprint is computed in terms of MCC value.

### Calculation of similarity

In order to compute similarity between two molecules, we calculated Tanimoto similarity score between two molecules using following equation

4 $$ {\mathrm{T}}_{\mathrm{s}}\left(\mathrm{X},\mathrm{Y}\right)=\frac{{\displaystyle {\sum}_{\mathrm{i}}\left({\mathrm{X}}_{\mathrm{i}}\wedge {\mathrm{Y}}_{\mathrm{i}}\right)}}{{\displaystyle {\sum}_{\mathrm{i}}\left({\mathrm{X}}_{\mathrm{i}}\vee {\mathrm{Y}}_{\mathrm{i}}\right)}} $$

Where T_s_ is the Tanimoto similarity score between compound *X* and *Y*; *X*_*i*_ and *Y*_*i*_ is fingerprint *i* of compound *X* and *Y*, respectively; *N* is total number of fingerprints. In this study, we computed two types of Tanimoto similarity scores called T_s1_ and T_s0_. The T_s1_ was calculated for fingerprint present (value 1) in the molecule and T_s0_ based upon the fingerprint absent (value 0) in the molecule.

### Potency score

The potency score of a query molecule was computed using following steps:

1. First, we computed Tanimoto similarity score T_s1_ between query compounds with each of anticancer molecules and selected highest T_s1_ called H^a^T_s1_.
2. Similarly, we also computed highest similarity score H^a^T_s0_ between the query and most similar anticancer molecules based on T_s0_.
3. Above steps were repeated to compute similarity scores H^n^T_s1_ and H^n^T_s0_ between the query and most similar non-anticancer molecule.
4. Finally, potency score was computed using following equation

5 $$ {P}_s= max\left({\mathrm{H}}^a{\mathrm{T}}_{\mathrm{s}1},{\mathrm{H}}^{\mathrm{a}}{\mathrm{T}}_{\mathrm{s}0}\right)\hbox{-} max\left({\mathrm{H}}^{\mathrm{n}}{\mathrm{T}}_{\mathrm{s}1},{\mathrm{H}}^{\mathrm{n}}{\mathrm{T}}_{\mathrm{s}0}\right) $$

Where *P*_*s*_ is the potency score of the query molecule and *max* is the maximum or highest score. If H^a^T_s1_ has q high score as compared to H^a^T_s0_, then it is the maximum score (*max*) of the anticancer molecule. Similarly, m*ax* score of non-anticancer molecules was selected based on the highest score of either H^n^T_s1_ or H^n^T_s0_. The advantage of using potency score instead of normal Tanimoto score is that it provides the structural similarity information of query molecule with anticancer, as well as with non-anticancer molecules.

### Frequency of functional groups

Functional groups were identified using the ChemmineR package of R [33]. The percent of compounds having specific functional groups was calculated using eq. 6. We also calculated the mean count of functional groups in compounds were compute using the eq. 7.

6 $$ {\mathrm{F}}_{\mathrm{G}}=\frac{{\displaystyle {\sum}_{\mathrm{i}=0}^{\mathrm{n}}}{\mathrm{P}}_{\mathrm{i}}^{\mathrm{j}}}{\mathrm{n}}\times 100 $$

7 $$ {\mathrm{M}}_{\mathrm{G}}=\frac{{\displaystyle {\sum}_{\mathrm{j}=1}^{\mathrm{n}}}{\mathrm{C}}_{\mathrm{i}}^{\mathrm{j}}}{\mathrm{n}} $$

Where M_G_ is the mean count of a functional group (G) in total number (n) of anticancer or non-anticancer compounds. C_i_^j^ is total count of a functional group (G) for the j^th^ compound with i value ranges from zero to maximum number of occurrence of functional group in a compound. The F_G_ is the mean frequency of a functional group (G) in total number (n) of anticancer or non-anticancer compounds with P_i_^j^ stands for presence or absence (value is either 0 or 1) of a functional group.

### Classification

For a comparison of potency score method with machine learning methods, we also developed models using various classifiers in WEKA package [34]. We also compare the performance of our method with SVM package [35]. For improving the overall performance, we developed the hybrid method by doing an average of the normalized potency score and SVM score. Since, the scale of potency score and SVM value are different, we normalized these values between −1.0 and 1.0.

### Performance evaluation

We have adopted the five-fold cross-validation technique to evaluate the performance of our models. In this technique, the compounds were randomly divided into five parts, where four parts were used for training and remaining part for testing. This process is carried out five times in such a way that each part was used once for testing. For obtaining unbiased results, the whole process of five-fold cross-validation was repeated 20 times. We report the final results as the average of 25-fold cross-validations. The performance of the method was assessed using various standard parameters like sensitivity, specificity, accuracy, and Matthews correlation coefficient (MCC) [36]. The receiver operating characteristic (ROC) graph was plotted using the ROCR package in R [37].

### Ethics

The study doesn’t involve any human, plant or animal subject. All the experiments were carried out using computational techniques.

## Results

### Frequency of functional groups

**Fig. 1:**
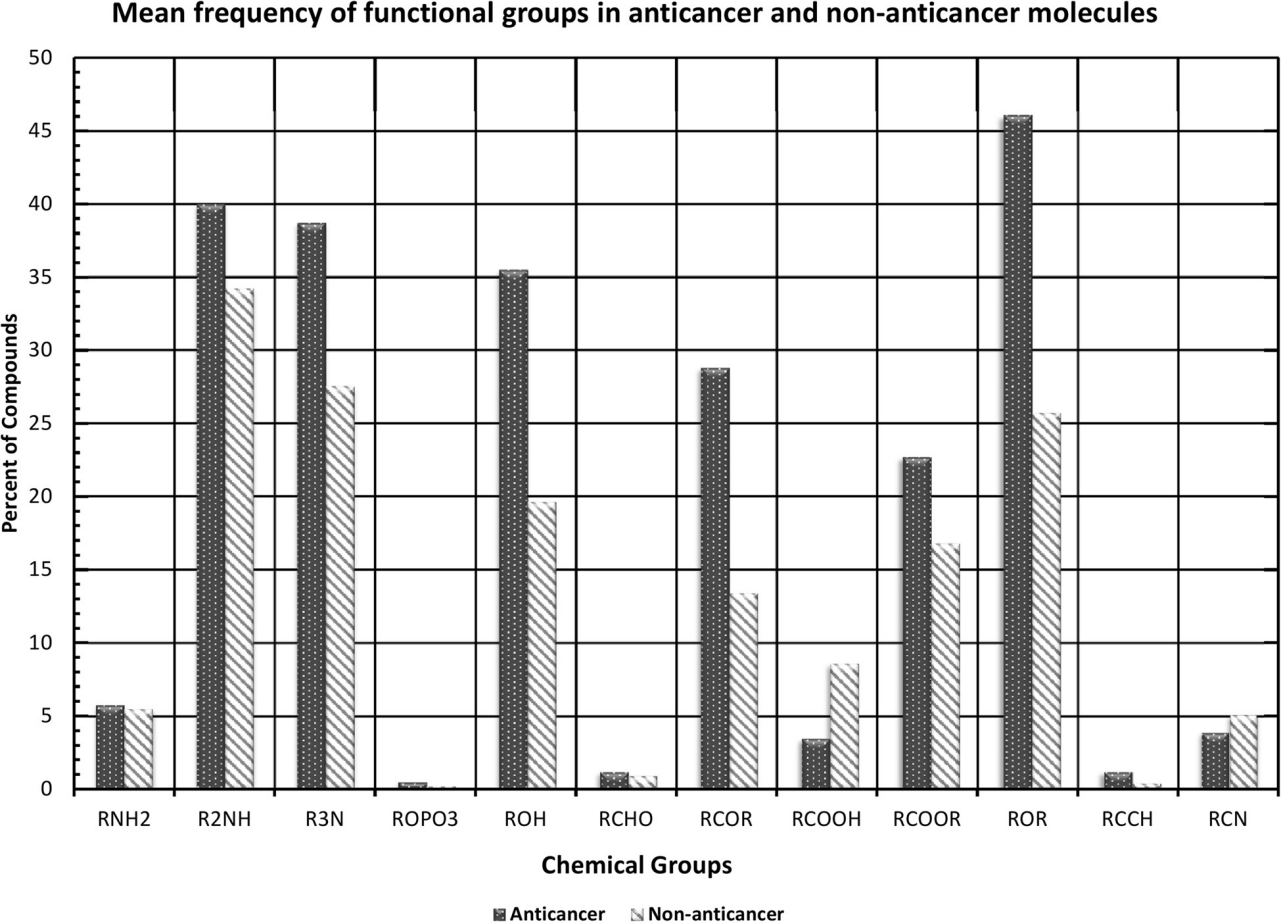
Functional groups present in anticancer and non-anticancer molecules along with their mean frequency

**Fig. 2:**
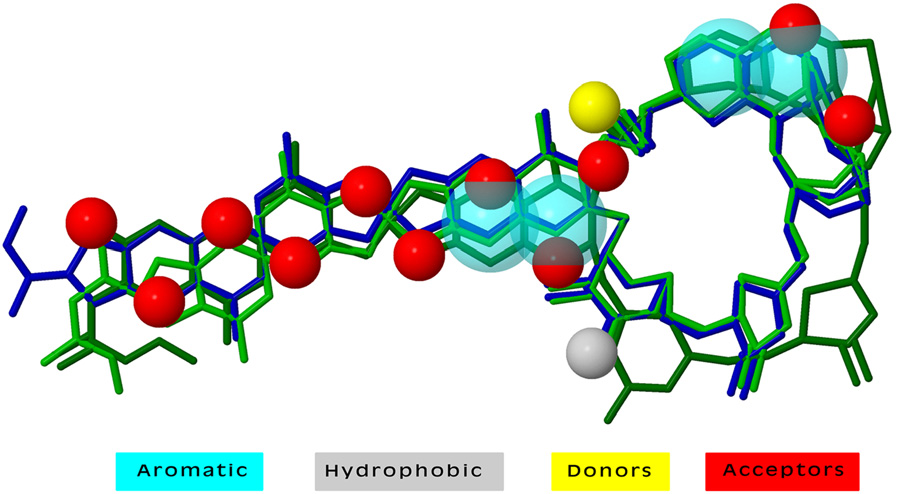
Pharmocophore alignment of most active anticancer molecules generated using PharmaGist

We tried to find out the predisposition of various functional groups in anticancer and non-anticancer molecules. Functional groups were identified using the ChemmineR package of R [33] and percentage of groups in compounds were computed using eq. 6. It was observed that certain functional groups (e.g., ROH, RCOR, RCOOR, ROR) have higher frequency and are predominantly present in anticancer molecules. These groups may be responsible for the anticancer activity of these active molecules as shown in Fig. 1. These functional groups can be further explored in designing of promiscuous anticancer molecules. We also calculated the total count of functional groups present in anticancer and non-anticancer molecules. It was observed that ROR group frequency range from 0 to 15 (maximum ROR was observed in a compound) as shown in Additional file 1: Figure S1. Further, we tried to find out the pharmacophore of most active molecules, which could be responsible for the anticancer activity. We aligned the top 20 molecules (in terms of activity) by PharmaGist software [38] and selected the most significant alignment (PharmaGist score of 77.61). This alignment identified total 18 features, which include twelve hydrogen bond acceptors, four aromatic, one hydrophobic and one hydrogen bond donor as shown in Fig. 2.

### Maximum common substructures (MCS)

**Fig. 3:**
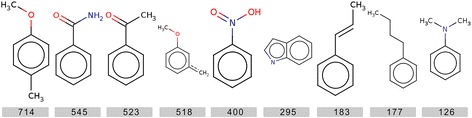
Maximum common substructures found in anticancer molecules along with the number of molecules having that particular substructure

We also determined the maximum common substructures in anticancer molecules using the LibMCS module of Chemaxon (http://www.chemaxon.com/). The analysis shown in Fig. 3 depicts the frequently occurring Maximum Common Substructures (MCS). The number beneath each MCS represents the total number of molecules in which that particular substructure was present according to MCS module. The 1st substructure is 1-methoxy-4-methylbenzene i.e., (methyl group is present at para position). The 2nd substructure is a part of known tyrosine kinase inhibitors like Imatinib and Nilotinib. The 6th substructure is indole structure, which is used for designing inhibitors against kinases especially EGFR [39]. The 3rd, 4th, 5th, 6th, 7th, 8th and 9th substructures are acetophenone, 1-methoxy benzene with partial double bond at meta position, nitrobenzene, indole, propenyl benzene, butyl benzene and dimethylaniline. We also calculate frequency of occurrences of these MCS in anticancer and non-anticancer compounds using substructure search option of jcsearch module of Chemaxon (Additional file 1: Table S1). The most popular common substructure 1-methoxy-4-methylbenzene found in 1115 (13.02 %) anticancer and 577 (5.89 %) non-anticancer (5.89 %) compound. Most of MCS have higher frequency in anticancer compounds as compare to non-anticancer compound.

### Analysis of fingerprints

In order to identify the best fingerprints, which are more abundant in anticancer or non-anticancer molecules, we used the MCC-based feature selection technique as described in Methods section. In brief MCC based feature selection involves two major steps; in first step the performance of each fingerprint is computed in terms of MCC; in 2nd step, fingerprints are ranked based on their MCC score [32]. In this study, we selected fingerprints having MCC score greater than 0.2 for the development of the model. It was observed that PubChem fingerprint number 12 is among the best fingerprints that can classify anticancer and non-anticancer molecules with an accuracy of 71.69 %. This fingerprint represents the presence of > = 16 carbon atoms in a compound. The best ten fingerprints along with their classification performance of anticancer and non-anticancer compounds are shown in Table 1. The detailed results of 126 fingerprints are given in Additional file 1: Table S2. It was observed that few CDK fingerprints are also efficient in distinguishing anticancer and non-anticancer molecules.

**Table 1:** The individual performance of best 10 selected fingerprints using MCC based approach

Best 10 fingerprints	Sensitivity	Specificity	Accuracy	MCC	FPR	AUC
PubchemFP12	79.3	65.1	71.69	0.45	0.48	0.72
ExtFP1013	52.5	85.7	70.19	0.41	0.65	0.69
ExtFP1012	78.4	61.9	69.61	0.41	0.47	0.7
PubchemFP192	58.4	79.4	69.6	0.39	0.61	0.69
GraphFP382	73.3	63.8	68.27	0.37	0.50	0.69
ExtFP1016	42	88.7	66.91	0.35	0.71	0.65
PubchemFP199	28.1	95.4	64.01	0.32	0.80	0.62
ExtFP1015	70.7	61.5	65.77	0.32	0.50	0.66
MACCSFP105	70.1	60.6	64.98	0.31	0.50	0.65
FP799	34.7	89.6	64.01	0.29	0.75	0.62

### Potency score based classification

In the current study, we compute the performance of models using five-fold cross validation technique with 20 runs as described by Li et. al. We selected the best fingerprints out of 9365 fingerprints for accurate, unbiased and quick development of classification method using MCC feature selection. First, we develop potency score based method using top 50 fingerprints having the highest MCC score. The best 50 fingerprints based method achieved 86.94 % accuracy with 0.74 MCC. Next, we developed method using best 100, 150 and 200 fingerprints and achieved 89.48 %, 90.1 %, 90.16 % accuracy respectively (Table 2). It was observed that using more than 150 fingerprints; there is no increase in performance of the method. Finally, we selected the fingerprints having MCC greater than 0.2 and obtained 126 fingerprints. We used these 126 fingerprints for developing prediction models and achieved 90.94 % accuracy with 0.82 MCC.

**Table 2:** The performance of potency score based method developed using different sets of fingerprints

Number of fingerprints	Sensitivity	Specificity	Accuracy	MCC	FPR	ROC
50	79.59	93.37	86.94	0.74	0.09	0.92
100	82.36	95.7	89.48	0.79	0.06	0.95
150	83.17	96.15	90.1	0.81	0.05	0.95
200	83.14	96.3	90.16	0.81	0.05	0.95
126	84.62	96.45	90.94	0.82	0.05	0.95

### Models based on machine learning techniques

In order to discriminate anticancer and non-anticancer molecules, we developed classification models using various machine learning techniques. The performance of models developed using different classifiers implemented in WEKA (i.e., Random forest, IBK, Naïve Bayes) and SVM^*light*^ [35, 40] has been shown in Table 3. The SVM-based models achieved highest accuracy 90.40 % with MCC 0.81 among all classifiers. The Random forest, IBK and Naïve Bayes based method achieved the highest accuracy in the range of 74.92–87.47 %. The models based on SVM and Random Forest achieved the best performance at the center of threshold and had broad range of MCC across various thresholds. The Random forest method achieved best performance using 100 trees; best SVM model trained using RBF kernel with parameter g = 0.1, c = 6 with j = 1; IBK method achieved best performance using kNN score of 3 with Manhattan distance algorithm.

**Table 3:** Comparative performance of models developed using 126 fingerprints at various thresholds has been shown in this table

	SVM			Random Forest		IBK		Naïve Bayes	
Threshold	Accuracy	MCC	Threshold	Accuracy	MCC	Accuracy	MCC	Accuracy	MCC
−1	72.40	0.54	0	46.63	0.00	46.63	0.00	46.63	0.00
−0.8	80.58	0.65	0.1	67.74	0.47	80.22	0.63	75.06	0.50
−0.6	84.72	0.72	0.2	77.73	0.61	82.59	0.67	74.99	0.50
−0.4	87.52	0.76	0.3	83.71	0.69	83.98	0.68	74.98	0.50
−0.2	89.60	0.79	0.4	86.33	0.73	85.71	0.72	74.92	0.50
0	90.40	0.81	0.5	87.47	0.75	85.31	0.71	74.86	0.49
0.2	90.24	0.80	0.6	86.47	0.73	85.10	0.71	74.79	0.49
0.4	89.00	0.79	0.7	83.81	0.69	82.73	0.67	74.79	0.49
0.6	86.35	0.74	0.8	79.03	0.62	81.22	0.65	74.81	0.49
0.8	82.65	0.68	0.9	71.86	0.51	80.90	0.65	74.77	0.49
1.0	70.49	0.48	1.0	59.30	0.28	80.87	0.65	73.37	0.48

### Performance of hybrid models

**Fig. 4:**
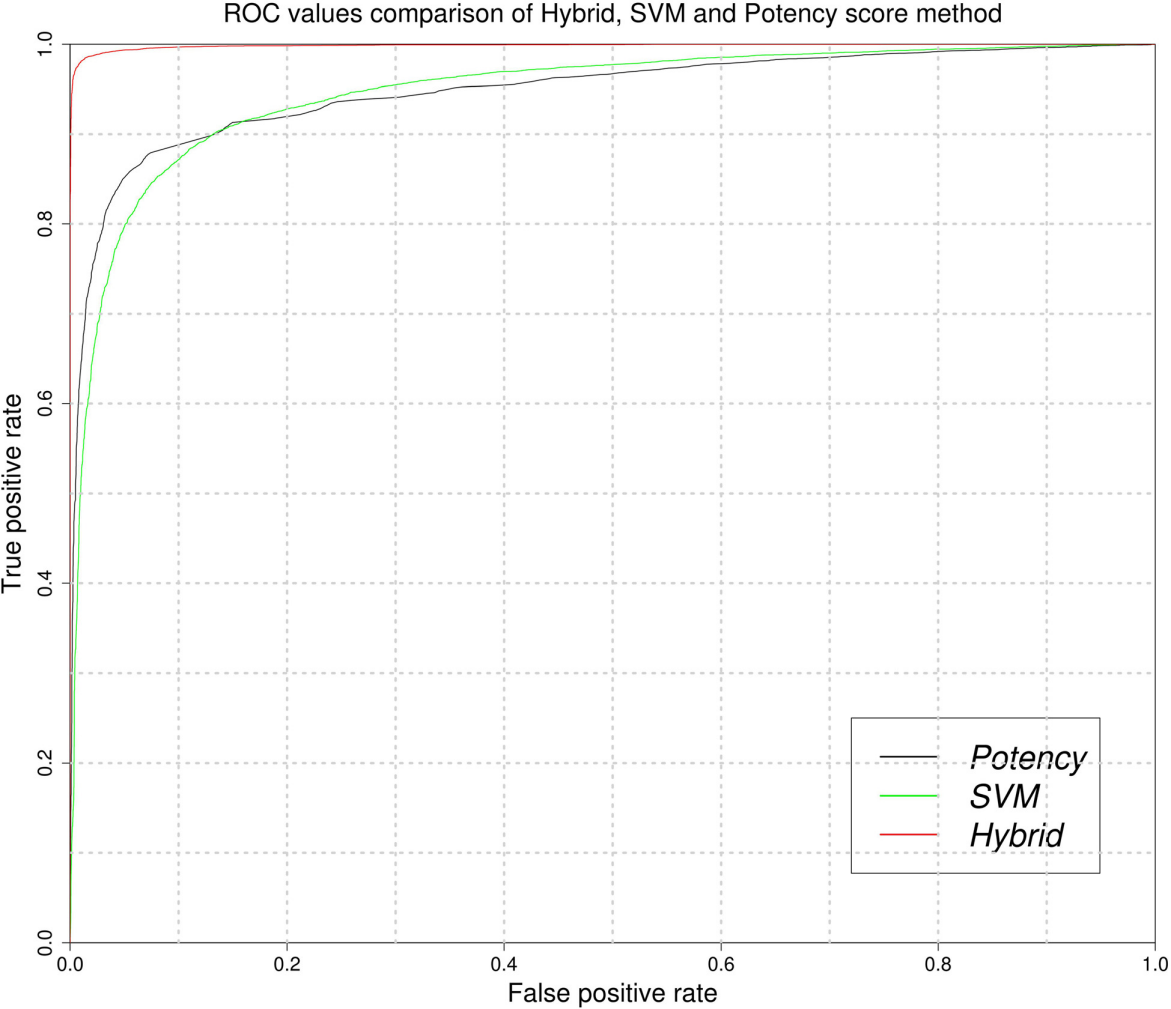
ROC plot of potency score, SVM and hybrid method developed using 126 fingerprints

As shown in both potency score based method and SVM-based model achieved maximum accuracy. The potency score method performs better, when query molecule is similar with anticancer molecules but perform poorly in case level of similarity is low. In case of SVM, the performance of the model is unaffected by similarity with known molecules. As shown in ROC curve at lower false positive rate (FPR), potency score performs better than SVM and at higher FPR, SVM perform better than potency score based method (Fig. 4). In order to take the advantage of potency score and SVM method, we developed the hybrid method. In case of hybrid method, first we compute SVM potency score of a query molecule and normalize these scores between −1.0 and 1.0. The average of normalize values is computed to obtain the hybrid score and used for predicting anticancer molecule. We developed a hybrid method using 126 best fingerprints and achieved highest MCC 0.85 with 0.98 AUC. The detail result of hybrid method are shown in Additional file 1: Table S3.

### Comparison with existing method

We compared the performance of our methods with existing method CDRUG. The CDRUG developed by Li et. al. achieved 65 %, 74 %, 81 % sensitivity at false positive rate (FPR) 0.05, 0.1, 0.2 respectively (Table 4). At 65 % sensitivity, both potency score method and SVM achieved 0.02 FPR and hybrid method achieved 0.01 FPR. As shown in Table 4, our models perform better than existing method CDRUG.

**Table 4:** Comparative performance of CDRUG (existing method) and our models based on potency score, SVM and hybrid approach

Method	Sensitivity	Specificity	Accuracy	MCC	FPR	AUC
CDRUG	65	-	-	-	0.05	0.88
	74				0.10	
	81	-	-	-	0.20	
Potency Score	65.8	98.9	83.5	0.7	0.02	0.95
	74.26	98.42	87.15	0.76	0.02	
	84.62	96.45	90.94	0.82	0.05	
SVM	65.47	98.66	83.34	0.69	0.02	0.95
	74.16	97.63	86.8	0.75	0.03	
	89.02	91.52	90.42	0.81	0.09	
Hybrid	65.57	99.63	83.75	0.71	0.01	0.98
	74.41	99.11	87.59	0.77	0.01	
	92.38	92.55	92.47	0.85	0.08	

### Description of the web server

In order to serve the scientific community, we developed a web server called “CancerIN” for predicting the anticancer potency of an unknown molecule and it’s GI_50_ across different cancer cell lines. This web server consists of three modules for designing, library screening and chemical analogs screening.

#### Draw molecule

**Fig. 5:**
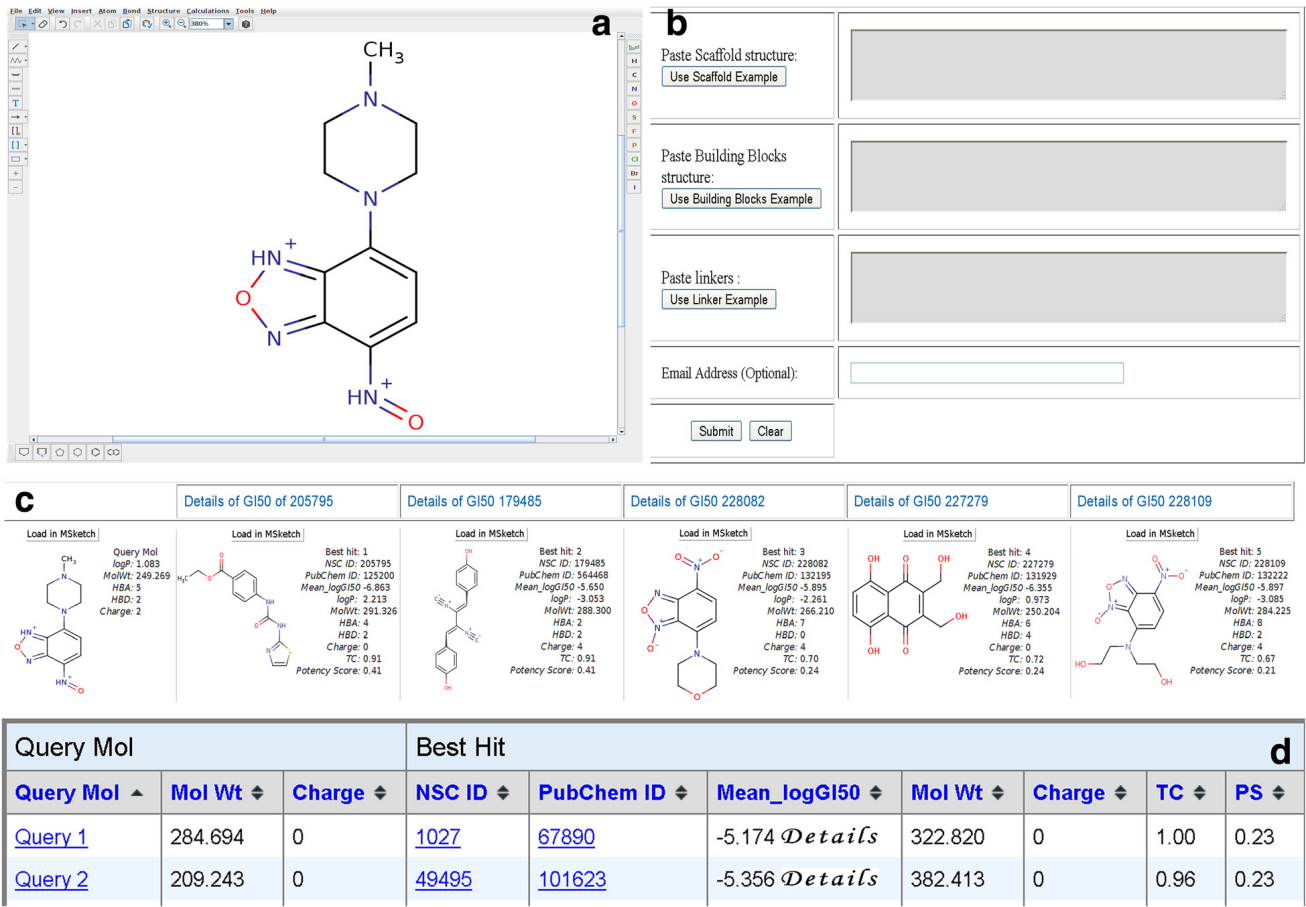
Various modules of CancerIN showing the input format and output display: **a** The Marvin draw applet for drawing molecules, **b** The input form for generation of analogs, **c** The output page of draw molecule module, and **d**. The result page of scan library showing the list of query molecules and the most similar anticancer molecules

This web server provides a user-friendly interface with options to draw a chemical compound using Marvin applet as shown in (Fig. 5a) [41]. The output consists of a 3D structure of query molecule with physicochemical properties and hybrid score. The five most similar anticancer molecules along with their NSC ID, PubChem ID, Mean_logGI_50_, Tanimoto similarity score, Potency score and physicochemical properties are also displayed. The details button provides the GI_50_ and LogGI_50_ score of similar molecule against different NCI-60 cancer cell lines. The user can select and further load either query molecule or any five similar molecules for further modifications based upon the structural similarity (Fig. 5c). The modified molecule can be further used as query molecule for increasing its potential anticancer activity.

#### Scan library

This web server also provides the provision to scan a chemical library in SMILES format [42]. The output consists of query molecule and five most similar anticancer molecules along with their other details as described above in tabular format (Fig. 5d).

#### Chemical analogs

We have also provided facility for the users to screen analogs generated from different combination of scaffold, building blocks and linkers using SmiLib [43] package (Fig. 5b) and subsequent prediction of their anticancer potency score. The results consist of query molecule and five most similar anticancer molecules in a tabular format.

#### Standalone

For the screening of thousands of molecules, we have developed CancerIN standalone, written in Python. The standalone version can screen thousands of molecules in less than 10 min. The input consists of a single file having chemical structures (SMILE format) of molecules for screening. The standalone version can be easily updated by replacing the underlying data file. The user can easily increase or decrease the number of fingerprints used for final prediction. The source code allows the scientific community to utilize the novel similarity-based method for prediction of various types of molecules.

In brief, the CancerIN web server predicts the anticancer capability of a single molecule, a library of chemicals or analogs. Since, our method also consider similarity, it also displays the GI_50_ of the similar anticancer molecule across different cancer cell lines. A careful analysis of the anticancer efficacy of five similar molecules aids in understanding the anticancer efficacy of query molecule against various cancer cell lines. The standalone version of CancerIN allows the users to scan a vast library of molecules for the screening of potential anticancer molecules. This standalone is available at CancerIN website http://crdd.osdd.net/oscadd/cancerin.

## Discussion and conclusion

The continuous development of novel anticancer drugs is imperative in order to tackle multi-drug resistance in cancer. At the same time, the development of an anticancer drug is very time-consuming, expensive and labor-intensive task. However, an integrated approach consisting of both computational and experimental approaches would be of great significance. Computational approaches are very helpful to identify or to narrow down potential lead molecules in a very short period without involving much money. Subsequently, the experimental approach may be used to validate these predictions. In this study, we developed QSAR models by considering the whole cell for anticancer activity for any class of molecules. The aim of the present study was to develop an efficient in silico method for screening of anticancer molecules against NCI-60 cancer cell lines. Thus, our method is a general method for predicting anticancer molecules irrespective of drug target or cell line. The performance of potency score method introduced in this study is comparable with models developed using machine-learning classifiers (e.g., Random forest, SVM, IBK and Naïve Bayes). We further improve the performance of our method by combining potency-score based model and SVM based method. In past, a method CDRUG has been developed on same dataset of chemicals for predicting anticancer molecules. Our best models outperform existing method CDRUG. Finally, we integrated these models in a web server for the betterment of scientific society working in this field.

## Additional files

Additional file 1: Figure S1. Counts of Functional groups present in anticancer and non-anticancer molecules. **Table S1.** Shows frequency of occurrence of MCS in anticancer and non-anticancer compounds according to LibMCS module of Chemaxon. Structures were search using jcsearch module of Chemaxon with substructure search option. **Table S2.** The individual performance of best 126 selected fingerprints using MCC based approach. **Table S3.** Performance of hybrid method developed using 126 fingerprints on different sensitivity. (DOC 356 kb)

## Abbreviations

AUC: area under the curve
FPR: false positive rate
MCC: Matthews Correlation Coefficient
MCS: maximum common substructures
MOA: mode of action
QSAR: quantitative structure-activity relationship
ROC: receiver operating characteristic
SVM: support vector machine
