# Supplementary material for: Prediction of anticancer molecules using hybrid model developed on molecules screened against NCI-60 cancer cell lines
Source: BMC Cancer. 2016 Feb 9;16:77. doi: 10.1186/s12885-016-2082-y (PMC4748564; doi:10.1186/s12885-016-2082-y)
Supplement: Additional file 1: Figure S1. — Counts of Functional groups present in anticancer and non-anticancer molecules. Table S1. Shows frequency of occurrence of MCS in anticancer and non-anticancer compounds according to LibMCS module of Chemaxon. Structures were search using jcsearch module of Chemaxon with substructure search option. Table S2. The individual performance of best 126 selected fingerprints using MCC based approach. Table S3. Performance of hybrid method developed using 126 fingerprints on different sensitivity. (DOC 356 kb) [file 12885_2016_2082_MOESM1_ESM.doc]

**Supplementary information page**

**Similarity based approach for predicting, screening and designing of anticancer molecules**

Harinder Singh, Rahul Kumar, Sandeep Singh, Kumardeep Chaudhary, Ankur Gautam and Gajendra P. S. Raghava*

Bioinformatics Centre, Institute of Microbial Technology, Sector 39-A, Chandigarh, India

*Corresponding author

Figure S1: Counts of Functional groups present in anticancer and non-anticancer molecules.


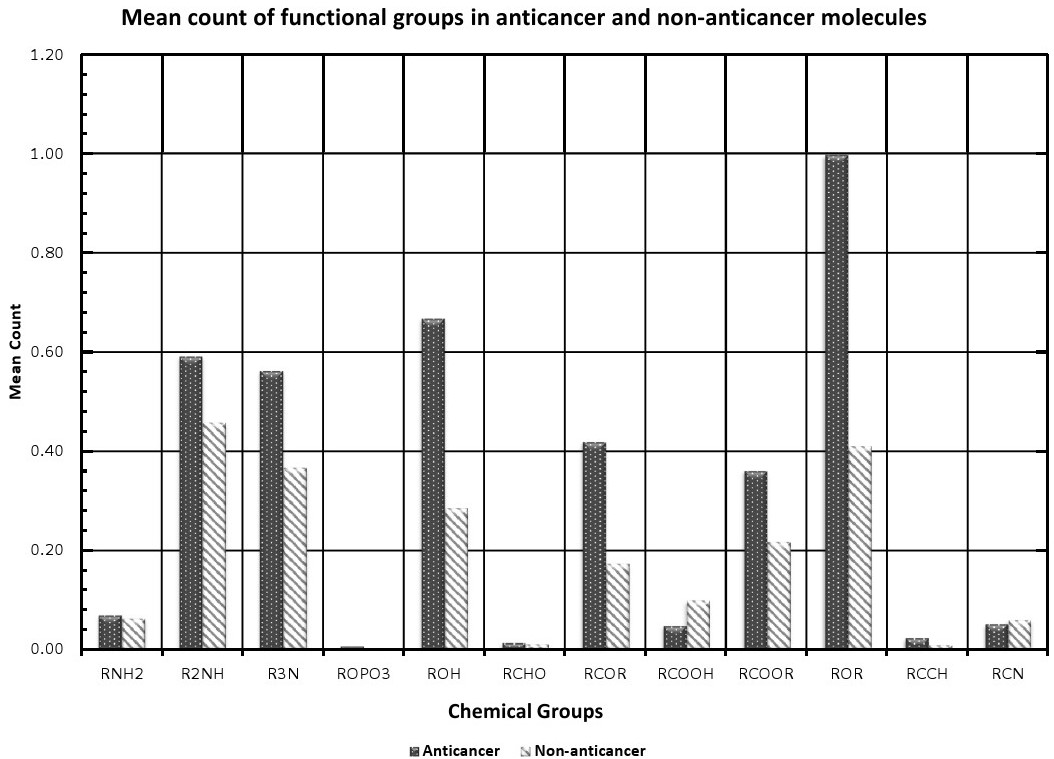


Table S1: Shows frequency of occurrence of MCS in anticancer and non-anticancer compounds according to LibMCS module of Chemaxon. Structures were search using jcsearch module of Chemaxon with substructure search option.

|  | LibMCS | Frequency of occurrence of MCS based on jcsearch | | | |
| --- | --- | --- | --- | --- | --- |
| MCS Number | Anticancer Compound | Anticancer Compounds | | Non-anticancer Compounds | |
|  | Count | Frequency | Percentage | Frequency | Percentage |
| 1 | 714 | 1115 | 13.02 | 577 | 5.89 |
| 2 | 545 | 594 | 6.94 | 486 | 4.96 |
| 3 | 523 | 680 | 7.94 | 496 | 5.06 |
| 4 | 518 | 558 | 6.51 | 130 | 1.33 |
| 5 | 400 | 556 | 6.49 | 580 | 5.92 |
| 6 | 295 | 405 | 4.73 | 101 | 1.03 |
| 7 | 183 | 655 | 7.65 | 290 | 2.96 |
| 8 | 177 | 826 | 9.64 | 469 | 4.78 |
| 9 | 126 | 542 | 6.33 | 479 | 4.89 |

Table S2: The individual performance of best 126 selected fingerprints using MCC based approach.

| [Fingerprint Description](http://crdd.osdd.net/oscadd/cancerin/bestdesc.php) | [PaDEL Fingerprint number](http://crdd.osdd.net/oscadd/cancerin/bestdesc.php) | [Sensitivity](http://crdd.osdd.net/oscadd/cancerin/bestdesc.php) | [Specificity](http://crdd.osdd.net/oscadd/cancerin/bestdesc.php) | [Accuracy](http://crdd.osdd.net/oscadd/cancerin/bestdesc.php) | [MCC](http://crdd.osdd.net/oscadd/cancerin/bestdesc.php) | [FPR](http://crdd.osdd.net/oscadd/cancerin/bestdesc.php) | [ROC](http://crdd.osdd.net/oscadd/cancerin/bestdesc.php) |
| --- | --- | --- | --- | --- | --- | --- | --- |
| PubchemFP12 | 3330 | 79.3 | 65.1 | 71.69 | 0.45 | 0.48 | 0.72 |
| ExtFP1013 | 2037 | 52.5 | 85.7 | 70.19 | 0.41 | 0.65 | 0.69 |
| ExtFP1012 | 2036 | 78.4 | 61.9 | 69.61 | 0.41 | 0.47 | 0.7 |
| PubchemFP192 | 3510 | 58.4 | 79.4 | 69.6 | 0.39 | 0.61 | 0.69 |
| GraphFP382 | 2509 | 73.3 | 63.8 | 68.27 | 0.37 | 0.5 | 0.69 |
| ExtFP1016 | 2040 | 42 | 88.7 | 66.91 | 0.35 | 0.71 | 0.65 |
| PubchemFP199 | 3517 | 28.1 | 95.4 | 64.01 | 0.32 | 0.8 | 0.62 |
| ExtFP1015 | 2039 | 70.7 | 61.5 | 65.77 | 0.32 | 0.5 | 0.66 |
| MACCSFP105 | 3256 | 70.1 | 60.6 | 64.98 | 0.31 | 0.5 | 0.65 |
| FP799 | 799 | 34.7 | 89.6 | 64.01 | 0.29 | 0.75 | 0.62 |
| PubchemFP189 | 3507 | 63.2 | 66.4 | 64.87 | 0.29 | 0.55 | 0.65 |
| GraphFP522 | 2649 | 48.3 | 78.9 | 64.61 | 0.29 | 0.65 | 0.64 |
| FP860 | 860 | 33.1 | 89.8 | 63.38 | 0.28 | 0.76 | 0.61 |
| PubchemFP696 | 4014 | 70.2 | 57.8 | 63.59 | 0.28 | 0.49 | 0.64 |
| GraphFP520 | 2647 | 79.6 | 46.7 | 62.03 | 0.28 | 0.4 | 0.63 |
| FP225 | 225 | 33.3 | 89.3 | 63.22 | 0.28 | 0.75 | 0.61 |
| FP204 | 204 | 38.5 | 85.6 | 63.64 | 0.28 | 0.72 | 0.62 |
| ExtFP1017 | 2041 | 23.2 | 95.9 | 62.02 | 0.28 | 0.83 | 0.6 |
| ExtFP724 | 1748 | 50.1 | 76.8 | 64.34 | 0.28 | 0.64 | 0.63 |
| ExtFP331 | 1355 | 44.1 | 81.5 | 64.07 | 0.28 | 0.68 | 0.63 |
| ExtFP302 | 1326 | 43.6 | 82.3 | 64.25 | 0.28 | 0.68 | 0.63 |
| GraphFP534 | 2661 | 51.6 | 74.5 | 63.81 | 0.27 | 0.62 | 0.63 |
| ExtFP968 | 1992 | 43.9 | 81.1 | 63.71 | 0.27 | 0.68 | 0.62 |
| FP952 | 952 | 33.6 | 88.1 | 62.65 | 0.26 | 0.75 | 0.61 |
| FP851 | 851 | 39.1 | 84.2 | 63.17 | 0.26 | 0.71 | 0.62 |
| FP475 | 475 | 54.7 | 71.1 | 63.49 | 0.26 | 0.6 | 0.63 |
| PubchemFP697 | 4015 | 54.1 | 71.9 | 63.58 | 0.26 | 0.6 | 0.63 |
| PubchemFP185 | 3503 | 81.3 | 42.9 | 60.83 | 0.26 | 0.38 | 0.62 |
| MACCSFP145 | 3296 | 81.6 | 42.4 | 60.66 | 0.26 | 0.37 | 0.62 |
| FP302 | 302 | 47.5 | 76.9 | 63.19 | 0.26 | 0.65 | 0.62 |
| GraphFP837 | 2964 | 38.2 | 84.3 | 62.81 | 0.26 | 0.72 | 0.61 |
| GraphFP554 | 2681 | 87.2 | 35.3 | 59.5 | 0.26 | 0.32 | 0.61 |
| GraphFP175 | 2302 | 62.1 | 63.9 | 63.06 | 0.26 | 0.54 | 0.63 |
| ExtFP766 | 1790 | 39.8 | 83.2 | 63 | 0.26 | 0.71 | 0.62 |
| ExtFP441 | 1465 | 46.3 | 78.1 | 63.27 | 0.26 | 0.66 | 0.62 |
| ExtFP340 | 1364 | 31.8 | 89 | 62.33 | 0.26 | 0.76 | 0.6 |
| FP76 | 76 | 66.5 | 58.3 | 62.08 | 0.25 | 0.5 | 0.62 |
| FP719 | 719 | 33.7 | 86.9 | 62.12 | 0.25 | 0.75 | 0.6 |
| FP704 | 704 | 44.9 | 78.3 | 62.73 | 0.25 | 0.67 | 0.62 |
| FP523 | 523 | 29.1 | 90.2 | 61.72 | 0.25 | 0.78 | 0.6 |
| FP453 | 453 | 40.9 | 81.6 | 62.61 | 0.25 | 0.7 | 0.61 |
| PubchemFP798 | 4116 | 31.4 | 89 | 62.16 | 0.25 | 0.76 | 0.6 |
| PubchemFP699 | 4017 | 38.4 | 83.4 | 62.41 | 0.25 | 0.71 | 0.61 |
| PubchemFP196 | 3514 | 25.6 | 92.8 | 61.49 | 0.25 | 0.81 | 0.59 |
| PubchemFP13 | 3331 | 13.4 | 99.1 | 59.12 | 0.25 | 0.89 | 0.56 |
| GraphFP892 | 3019 | 65.3 | 59.9 | 62.43 | 0.25 | 0.51 | 0.63 |
| GraphFP717 | 2844 | 33.4 | 87.5 | 62.24 | 0.25 | 0.75 | 0.6 |
| GraphFP465 | 2592 | 29.7 | 90.4 | 62.06 | 0.25 | 0.78 | 0.6 |
| GraphFP319 | 2446 | 52.4 | 72.5 | 63.1 | 0.25 | 0.61 | 0.62 |
| GraphFP286 | 2413 | 89.4 | 31.5 | 58.47 | 0.25 | 0.29 | 0.6 |
| ExtFP920 | 1944 | 26.8 | 92 | 61.62 | 0.25 | 0.8 | 0.59 |
| ExtFP511 | 1535 | 29.7 | 89.7 | 61.74 | 0.25 | 0.78 | 0.6 |
| ExtFP424 | 1448 | 61.3 | 63.3 | 62.37 | 0.25 | 0.54 | 0.62 |
| ExtFP292 | 1316 | 36.1 | 85.4 | 62.43 | 0.25 | 0.73 | 0.61 |
| ExtFP283 | 1307 | 26.2 | 92.1 | 61.36 | 0.25 | 0.8 | 0.59 |
| ExtFP187 | 1211 | 35.1 | 86.1 | 62.29 | 0.25 | 0.74 | 0.61 |
| FP893 | 893 | 43.4 | 79.1 | 62.45 | 0.24 | 0.68 | 0.61 |
| FP866 | 866 | 30.7 | 89 | 61.79 | 0.24 | 0.77 | 0.6 |
| FP744 | 744 | 49.7 | 73.5 | 62.4 | 0.24 | 0.63 | 0.62 |
| FP727 | 727 | 39.6 | 81.7 | 62.1 | 0.24 | 0.7 | 0.61 |
| PubchemFP688 | 4006 | 87.2 | 33.6 | 58.64 | 0.24 | 0.31 | 0.6 |
| PubchemFP341 | 3659 | 68 | 56.3 | 61.76 | 0.24 | 0.49 | 0.62 |
| MACCSFP152 | 3303 | 68 | 56.3 | 61.76 | 0.24 | 0.49 | 0.62 |
| GraphFP819 | 2946 | 56.9 | 67.4 | 62.5 | 0.24 | 0.58 | 0.62 |
| GraphFP791 | 2918 | 72.5 | 50.6 | 60.81 | 0.24 | 0.44 | 0.62 |
| GraphFP462 | 2589 | 26.9 | 91.5 | 61.35 | 0.24 | 0.8 | 0.59 |
| GraphFP411 | 2538 | 55.5 | 68.4 | 62.4 | 0.24 | 0.59 | 0.62 |
| FP193 | 193 | 42.1 | 80.3 | 62.51 | 0.24 | 0.69 | 0.61 |
| ExtFP409 | 1433 | 42.6 | 79.2 | 62.16 | 0.24 | 0.68 | 0.61 |
| ExtFP405 | 1429 | 40.8 | 81.3 | 62.42 | 0.24 | 0.7 | 0.61 |
| ExtFP299 | 1323 | 28.4 | 90.1 | 61.31 | 0.24 | 0.78 | 0.59 |
| ExtFP221 | 1245 | 30 | 89.3 | 61.67 | 0.24 | 0.77 | 0.6 |
| ExtFP161 | 1185 | 37.4 | 84 | 62.28 | 0.24 | 0.72 | 0.61 |
| ExtFP138 | 1162 | 35.2 | 85.6 | 62.13 | 0.24 | 0.74 | 0.6 |
| ExtFP76 | 1100 | 56.5 | 67.2 | 62.21 | 0.24 | 0.58 | 0.62 |
| FP766 | 766 | 28.3 | 89.3 | 60.89 | 0.23 | 0.78 | 0.59 |
| FP764 | 764 | 55.2 | 68 | 62.02 | 0.23 | 0.59 | 0.62 |
| FP589 | 589 | 46.6 | 75.5 | 62.02 | 0.23 | 0.65 | 0.61 |
| PubchemFP735 | 4053 | 26.4 | 91 | 60.86 | 0.23 | 0.8 | 0.59 |
| MACCSFP125 | 3276 | 71.2 | 51.3 | 60.57 | 0.23 | 0.45 | 0.61 |
| GraphFP684 | 2811 | 60.3 | 62.3 | 61.37 | 0.23 | 0.54 | 0.61 |
| FP274 | 274 | 38.2 | 82.5 | 61.83 | 0.23 | 0.71 | 0.6 |
| GraphFP320 | 2447 | 30.4 | 88.2 | 61.24 | 0.23 | 0.77 | 0.59 |
| GraphFP298 | 2425 | 69.4 | 53.4 | 60.87 | 0.23 | 0.47 | 0.61 |
| GraphFP31 | 2158 | 51.5 | 70.8 | 61.82 | 0.23 | 0.61 | 0.61 |
| ExtFP1011 | 2035 | 93 | 24.2 | 56.31 | 0.23 | 0.23 | 0.59 |
| FP173 | 173 | 32.1 | 86.8 | 61.31 | 0.23 | 0.76 | 0.59 |
| ExtFP659 | 1683 | 29.4 | 89.1 | 61.27 | 0.23 | 0.78 | 0.59 |
| ExtFP652 | 1676 | 54.6 | 68.5 | 62.01 | 0.23 | 0.59 | 0.62 |
| ExtFP634 | 1658 | 33 | 86 | 61.27 | 0.23 | 0.75 | 0.59 |
| ExtFP611 | 1635 | 50 | 72.3 | 61.89 | 0.23 | 0.62 | 0.61 |
| ExtFP597 | 1621 | 35.8 | 84.5 | 61.77 | 0.23 | 0.73 | 0.6 |
| ExtFP591 | 1615 | 42.6 | 78.8 | 61.89 | 0.23 | 0.68 | 0.61 |
| ExtFP416 | 1440 | 38 | 82.7 | 61.87 | 0.23 | 0.71 | 0.6 |
| ExtFP391 | 1415 | 31.3 | 87.3 | 61.2 | 0.23 | 0.76 | 0.59 |
| ExtFP386 | 1410 | 33.3 | 86.3 | 61.58 | 0.23 | 0.75 | 0.6 |
| ExtFP369 | 1393 | 43.7 | 78 | 62.05 | 0.23 | 0.67 | 0.61 |
| ExtFP350 | 1374 | 47.6 | 74.6 | 62 | 0.23 | 0.64 | 0.61 |
| FP130 | 130 | 28.6 | 89.3 | 60.99 | 0.23 | 0.78 | 0.59 |
| ExtFP193 | 1217 | 31.4 | 87.2 | 61.16 | 0.23 | 0.76 | 0.59 |
| ExtFP80 | 1104 | 33.1 | 86.4 | 61.52 | 0.23 | 0.75 | 0.6 |
| FP1015 | 1015 | 49.8 | 73 | 62.19 | 0.23 | 0.63 | 0.61 |
| FP988 | 988 | 34.4 | 84.4 | 61.06 | 0.22 | 0.74 | 0.59 |
| FP922 | 922 | 34 | 85.1 | 61.26 | 0.22 | 0.74 | 0.6 |
| KRFP2986 | 7491 | 43.3 | 77.2 | 61.39 | 0.22 | 0.67 | 0.6 |
| FP533 | 533 | 27.9 | 89.6 | 60.81 | 0.22 | 0.79 | 0.59 |
| FP444 | 444 | 33.1 | 85.2 | 60.9 | 0.22 | 0.75 | 0.59 |
| FP438 | 438 | 29.9 | 87.8 | 60.81 | 0.22 | 0.77 | 0.59 |
| FP410 | 410 | 39.4 | 80.9 | 61.56 | 0.22 | 0.7 | 0.6 |
| FP325 | 325 | 20.4 | 94 | 59.67 | 0.22 | 0.84 | 0.57 |
| MACCSFP99 | 3250 | 43.4 | 77.2 | 61.4 | 0.22 | 0.67 | 0.6 |
| GraphFP893 | 3020 | 24 | 91.9 | 60.25 | 0.22 | 0.81 | 0.58 |
| GraphFP731 | 2858 | 32.2 | 85.9 | 60.86 | 0.22 | 0.75 | 0.59 |
| GraphFP455 | 2582 | 42.6 | 77.6 | 61.28 | 0.22 | 0.68 | 0.6 |
| GraphFP318 | 2445 | 47.4 | 73.8 | 61.5 | 0.22 | 0.64 | 0.61 |
| GraphFP255 | 2382 | 51.9 | 69.8 | 61.43 | 0.22 | 0.61 | 0.61 |
| ExtFP883 | 1907 | 33.3 | 85.3 | 61.05 | 0.22 | 0.75 | 0.59 |
| ExtFP752 | 1776 | 40.3 | 79.9 | 61.41 | 0.22 | 0.69 | 0.6 |
| ExtFP530 | 1554 | 34.7 | 84.3 | 61.18 | 0.22 | 0.74 | 0.6 |
| ExtFP408 | 1432 | 45.1 | 75.9 | 61.52 | 0.22 | 0.66 | 0.6 |
| ExtFP399 | 1423 | 31.5 | 86.9 | 61.04 | 0.22 | 0.76 | 0.59 |
| FP139 | 139 | 53.9 | 68.1 | 61.45 | 0.22 | 0.59 | 0.61 |
| ExtFP238 | 1262 | 30.2 | 87.6 | 60.8 | 0.22 | 0.77 | 0.59 |
| ExtFP160 | 1184 | 31 | 86.9 | 60.83 | 0.22 | 0.76 | 0.59 |
| ExtFP102 | 1126 | 36.3 | 83.1 | 61.25 | 0.22 | 0.72 | 0.6 |
| ExtFP95 | 1119 | 27.1 | 90 | 60.64 | 0.22 | 0.79 | 0.59 |

Table S3: Performance of hybrid method developed using 126 fingerprints on different sensitivity.

| **Threshold** | **Sensitivity** | **Specificity** | **Accuracy** | **MCC** | **FPR** |
| --- | --- | --- | --- | --- | --- |
| -1 | 100 | 0 | 46.63 | 0 | 0.53 |
| -0.9 | 100 | 0.01 | 46.63 | 0.01 | 0.53 |
| -0.8 | 100 | 0.1 | 46.68 | 0.02 | 0.53 |
| -0.7 | 100 | 0.64 | 46.97 | 0.05 | 0.53 |
| -0.6 | 99.99 | 3.6 | 48.55 | 0.13 | 0.52 |
| -0.5 | 99.94 | 19.4 | 56.96 | 0.32 | 0.48 |
| -0.4 | 99.65 | 40.95 | 68.32 | 0.49 | 0.4 |
| -0.3 | 98.94 | 62.91 | 79.71 | 0.65 | 0.3 |
| -0.2 | 97.59 | 76.98 | 86.59 | 0.75 | 0.21 |
| -0.1 | 95.52 | 86.49 | 90.7 | 0.82 | 0.14 |
| 0 | 92.38 | 92.55 | 92.47 | 0.85 | 0.08 |
| 0.1 | 87.74 | 96.2 | 92.26 | 0.85 | 0.05 |
| 0.2 | 80.59 | 98.32 | 90.05 | 0.81 | 0.02 |
| 0.3 | 68.9 | 99.48 | 85.22 | 0.73 | 0.01 |
| 0.4 | 47.99 | 99.92 | 75.71 | 0.57 | 0 |
| 0.5 | 25.18 | 99.99 | 65.11 | 0.39 | 0 |
| 0.6 | 8.99 | 100 | 57.56 | 0.22 | 0 |
| 0.7 | 2.39 | 100 | 54.49 | 0.11 | 0 |
| 0.8 | 0.49 | 100 | 53.6 | 0.05 | 0 |
| 0.9 | 0.08 | 100 | 53.41 | 0.02 | 0 |
| 1 | 0.01 | 100 | 53.38 | 0.01 | 0 |
